# Response of the Human Milk Microbiota to a Maternal Prebiotic Intervention Is Individual and Influenced by Maternal Age

**DOI:** 10.3390/nu12041081

**Published:** 2020-04-13

**Authors:** Marina Padilha, Asker Brejnrod, Niels Banhos Danneskiold-Samsøe, Christian Hoffmann, Julia de Melo Iaucci, Vanessa Pereira Cabral, Douglas Xavier-Santos, Carla Romano Taddei, Karsten Kristiansen, Susana Marta Isay Saad

**Affiliations:** 1 School of Pharmaceutical Sciences, University of São Paulo, São Paulo 05508-000, Brazil; c.hoffmann@usp.br (C.H.); juiaucci@gmail.com (J.d.M.I.); vanessapcabral@gmail.com (V.P.C.); tecnol.douglas@rocketmail.com (D.X.-S.); crtaddei@usp.br (C.R.T.); 2 Food Research Center (FoRC), University of São Paulo, São Paulo 05508-000, Brazil; 3 Laboratory of Genomics and Molecular Biomedicine, Department of Biology, University of Copenhagen, DK-2100 Copenhagen, Denmark; askerbrejnrod@gmail.com (A.B.); nds@bio.ku.dk (N.B.D.-S.); kk@bio.ku.dk (K.K.); 4 School of Public Health, University of São Paulo, São Paulo 01246-904, Brazil; 5 Faculty of High Education of the Interior of São Paulo, Marília 17512-130, Brazil; 6 School of Arts, Sciences and Humanities, University of São Paulo, São Paulo 03828-000, Brazil

**Keywords:** fructooligosaccharide, breast milk, breastfeeding, infant, clinical intervention, microorganisms

## Abstract

Maternal bacteria are shared with infants via breastfeeding. Prebiotics modulate the gut microbiota, promoting health benefits. We investigated whether the maternal diet supplementation with a prebiotic (fructooligosaccharides, FOS) could influence the milk microbiota. Twenty-eight lactating women received 4.5 g of fructooligosaccharides + 2 g of maltodextrin (FOS group) and twenty-five received 2 g of maltodextrin (placebo group) for 20 days. Breast-milk samples were taken before and after the intervention. The DNA from samples was used for 16S rRNA sequencing. No statistical differences between the groups were found for the bacterial genera after the intervention. However, the distances of the trajectories covered by paired samples from the beginning to the end of the supplementation were higher for the FOS group (*p* = 0.0007) indicating greater changes in milk microbiota compared to the control group. Linear regression models suggested that the maternal age influenced the response for FOS supplementation (*p* = 0.02). Interestingly, the pattern of changes to genus abundance upon supplementation was not shared between mothers. We demonstrated that manipulating the human milk microbiota through prebiotics is possible, and the maternal age can affect this response.

## 1. Introduction

The gut microbiota has been shown to play an important role in human health [1,2]. The benefits of the interaction with microorganisms are particularly related to the development of the immune system, prevention against pathogens, and protection against diseases, such as allergies, type 1 diabetes, and obesity [3,4,5,6].

The human gut colonization by microorganisms mainly begins at birth. As soon as the baby is born, it is exposed to a maternal and environmental microbial community which will shape the newborn’s gut microbiota [7,8]. After birth, dietary practices including formula or breast feeding and use of antibiotics have been linked to the assembly of the gut microbiota structure [8,9].

Maternal-neonate microbial transfers have been suggested to be evolutionary and have been preserved in several species of the animal kingdom [10]. They can occur during delivery, by the birth canal [11], and via breastfeeding [12]. Bäckhed et al. [11] demonstrated that vaginally delivered newborns have a more similar gut bacterial profile to their mothers’ gut, when compared to C-section-delivered newborns. The maternal gut has been proposed as providing part of the microbial community transferred to vaginally delivered newborns, possibly due to the proximity of the maternal gut and the birth canal. In addition, Asnicar et al. [2] identified that specific strains, including *Bifidobacterium bifidum*, *Coprococcus comes*, and *Ruminococcus bromii*, were present in breast milk and infant feces from the same mother–infant pair, while being distinct from those carried by other pairs, indicating a vertical transmission via breastfeeding. 

Certain bacteria, in particular species of *Bifidobacterium* and *Lactobacillus*, are strongly related to benefits in infant and adult health [13]. These bacteria can be stimulated by the presence of specific compounds, namely prebiotics [14]. By definition, a prebiotic is “a substrate that is selectively utilized by host microorganisms conferring a health benefit” [15]. The most studied prebiotics in infants, pregnant and lactating women are fructooligosaccharides (FOS) and galactooligosaccharides (GOS) [16,17]. These oligosaccharides are non-digestible by humans and can be used as substrates by specific bacteria in order to obtain energy [18,19].

Given that prebiotics can be utilized by host microorganisms, promoting benefits to host health, we investigated whether the maternal diet supplementation with prebiotics (fructooligosaccharides) could influence the dynamics of the human milk microbiota. To our knowledge, this is the first study to address this issue.

## 2. Materials and Methods

### 2.1. Subject and Study Design

Ninety-two healthy lactating women of 18–37 years of age with uncomplicated pregnancies and who had vaginal deliveries at the University Hospital of the University of São Paulo (SP, Brazil) were enrolled in the study, which was conducted between September 2014 and June 2016.

Inclusion criteria involved having no chronic gastrointestinal disease, genetic disease, cardiac disease, kidney disease, hypertension, diabetes mellitus or immunodeficiency diseases, eclampsia or gestational diabetes during pregnancy, mastitis during the lactation period, as well as having term babies (born between 37 and 42 weeks), with adequate weight for gestational age, having normal bowel frequency (minimum once every 2 days, maximum 3 times per day), and carrying out breastfeeding. Participants were required not to have taken proton pump inhibitors, H2 receptor antagonists, antidepressants, narcotics, anticholinergic medications, laxatives, or anti-diarrhea medications within 30 days prior to collection the milk samples, and a regular consumption of commercially available prebiotic- or probiotic-supplemented product. The volunteers were screened through medical records and from questionnaires.

The study was performed as a single-blinded randomized placebo-controlled trial. The FOS group participants received 20 sachets containing 4.5 g of the prebiotic fructooligosaccharides (FOS, Beneo P95, Orafti, Oreye, Belgium) + 2 g of maltodextrin (Nidex, Nestlé, Araçatuba, SP, Brazil). The placebo group participants received 20 sachets containing only 2 g of maltodextrin. Since the total powder amount in each sachet of the FOS group and the placebo group was noticeably different, the researcher could not be blinded and only the participants were blinded in this trial. The volunteers were instructed to dissolve all the powder content of one sachet in approximately 200 mL of water once a day for 20 days, starting at the day after the first meeting and ending at the day before the second meeting. Detailed instructions were described on the label of each sachet. The volunteers were also asked to maintain their habitual diet throughout the intervention period. Written informed consent was obtained from all participants. The study protocol was conducted according to the guidelines laid down in the Declaration of Helsinki and approved by the Research Ethics Committee of the School of Pharmaceutical Sciences, University of São Paulo, São Paulo, Brazil and by the Research Ethics Committee of the University Hospital of the University of São Paulo, São Paulo, Brazil-CAAE: 27247614.6.0000.0067 (Brazilian Registry of Clinical Trials RBR-3r2kv9).

### 2.2. Data Collection

Information on pregnancy, family income, bowel frequency, alcohol consumption, smoking, pre-gestational and perinatal anthropometric data (weight and height), and information about the newborn were obtained by a structured questionnaire applied in a face-to-face interview before the clinical trial. Two 24-hour food recalls (24-HR) were performed, at day 30 (± 4) after delivery and 21 days later, immediately after the consumption of the last sachet of the intervention in order to monitor the volunteer diet before and after the trial. Dietary data were analyzed by Dietpro® software (Dietpro, Viçosa, MG, Brazil) to obtain the estimated nutrient intake. Data on complaints about bowel behavior (e.g., abdominal pain, distension, flatulence) were registered before and after the intervention.

In addition, the volunteers were contacted by telephone at day 10 (in the middle of the intervention period) to promote compliance and to assess the occurrence of side effects and complaints. We considered the adherence for women who had consumed at least 90% of the sachets (18 sachets), which was checked by asking the number of returned sachets at the end of the study.

### 2.3. Milk Samples Collection

Milk samples were collected from volunteers before [at 30 (± 4) days after delivery] and immediately after the intervention. For collection of milk samples, the nipple and surrounding area were cleaned with 1% chlorhexidine to reduce the presence of skin bacteria. Next, milk was collected by manual pressure into a sterile tube according to Martín et al. [20] and Jost et al. [21]. The first drops (approximately 200 µL) were discarded. All the samples were kept on ice for 4 hours; the samples were aliquoted and stored at –80 °C for later DNA extraction.

### 2.4. DNA Isolation

Human milk samples (1.5 mL) were centrifugated at 15,700 g for 15 minutes to separate pellet prokaryotic cells. The pellets were resuspended in 1000 µL of Tris EDTA (10 mM Tris-HCl [pH 7.5] and 1 mM EDTA [pH 7.6]) buffer. The suspension was centrifuged at 15,700 g for 15 min. The samples were lysed in 200 µL of TELS (20 mg/mL) lysozyme:1 M Tris-HCl [pH 7.5], 0.5 M EDTA [pH 8.0], 20% sucrose) buffer; then, it was incubated for 60 min at 37 °C. The DNA isolation proceeded using the QIAamp DNA Mini Kit (QIAGEN, Hilden, Germany) according to the manufacturer’s protocol for Gram-positive bacteria. 

DNA quality and concentrations were determined using a Nanodrop ND-1000 (Thermo Fisher Scientific, Waltham, MA, USA).

### 2.5. PCR Amplification for Sequencing

The genomic DNA isolated from the clinical samples was amplified by a nested PCR. For the first round of amplifications, the primers 341F (CCTAYGGGRBGCASCAG) and 806R (GGACTACHVGGGTWTCTAAT), published by YU et al. [22], were used. For the second round, barcoded primers that amplify the V4 hypervariable region of the 16S ribosomal RNA (16S rRNA) were used. The primers pair 2 are described as follows:

Tagged 515F (AATGATACGGCGACCACCGAGATCTACAC NNNNNNNN GT GTGCCAGCMGCCGCGGTAA).

Tagged 806R (CAAGCAGAAGACGGCATACGAGAT NNNNNNNNNNNN AGTCAGTCAG CC GGACTACHVGGGTWTCTAAT).

Where “N” indicates the nucleotides of the barcode sequence. Both primers with Illumina adaptor had sequences at the 5’ end [23].

The PCR reactions were carried out in a 25 μL mixture (final volume), containing 500 nM (for the first round) or 200 nM (for the second round) of each primer pair, 0.2 mM dNTPs (Thermo Fisher Scientific), 0.5 units Phusion high fidelity DNA polymerase (Thermo Fisher Scientific), 1 x Phusion Green HF buffer (Thermo Fisher Scientific), and 10 µL of the DNA sample (for the first round) or 2 µL of PCR product from the first amplification (for the second round). Thermal cycling was carried out in a Veriti® Thermal cycler (Applied Biosystem, Thermo Fisher Scientific) under the following conditions:

98 °C for 30 s followed by “X” cycles of 98 °C for 5 s, 56 °C for 20 s, and 72 °C for 20 s, wherein “X” was 40 or 15 for the first and second round of amplification, respectively.

After amplification, the PCR products were purified using the Agencourt AMPure XP purification system (Beckman Coulter, Danvers, MA, USA). The amplicon concentration was measured with the PicoGreen kit (Thermo Fisher Scientific). Equimolar amounts of each PCR product were then pooled together and sequenced using an Illumina MiSeq V2 PE500 cartridge (500 cycles) on an Illumina MiSeq (Illumina®, San Diego, CA, USA).

### 2.6. Sequence Processing

Initial analysis was carried out using the Quantitative Insights Into Microbial Ecology (QIIME) pipeline v1.9.1 with default settings. Chimera checking was performed using UCHIME69 and de novo operational taxonomic units (OTU)-picking was performed using UCLUST70 with 97% sequence similarity. Representative sequences were assigned taxonomy against the Greengenes database v13_871 using the Ribosomal Database Project - classifier [24]. Subsequent analyses were performed with the R version 3.4.3 using the metagenomeSeq [25], phyloSeq [26], vegan [27], and ggplot2 [28] packages. Data were filtered for low abundance of operational taxonomic units (OTUs) by removal of OTUs present in fewer than 3 of all the samples and with a relative abundance higher than 0.5% across all OTUs. Analyses in R were performed with sequences per sample after filtering. Statistical analysis was performed on filtered data based on effective sample sizes, where samples were not included if they had fewer than 10,000 or more than 100,000 OTUs.

### 2.7. Data Deposition and Materials Sharing

Sequence data have been deposited in the National Center for Biotechnology Information (NCBI) under the project number PRJNA479106 (https://www.ncbi.nlm.nih.gov/Traces/study/?acc = SRP151896) and are available under SRA accession SRP151896.

### 2.8. Quantitative PCR 

Quantitative PCR (qPCR) was employed for the quantification of *Bifidobacterium* spp. and *Lactobacillus* spp. from human milk samples collected before and after 20 days of supplementation. The qPCR was performed using Taqman (ThermoFisher Scientific) or SYBR green (ThermoFisher Scientific) for *Bifidobacterium* spp. and *Lactobacillus* spp., respectively. 

For *Bifidobacterium* spp., the amplifications were carried out in a 25 µL mixture (final volume) containing 12.5 µL of TaqMan® Universal PCR 2X Master Mix (ThermoFisher Scientific), 200 nM of each primer (F_Bifid 09c CGG GTG AGT AAT GCG TGA CC, R_Bifid 06 TGA TAG GAC GCG ACC CCA [29]), 250 nM of probe (P_Bifid 6FAM-CTC CTG GAA ACG GGT G [29]), and 5 µL of the DNA template. Reactions were performed under the following conditions: 1 cycle at 95 ºC for 10 minutes, followed by 40 cycles at 95 ºC for 30 seconds, and at 60 °C for 1 minute.

For *Lactobacillus* spp., the qPCR reactions were carried out in a 25 μL mixture (final volume), containing 12.5 µL of SYBR Green® PCR 2X Master Mix (ThermoFisher Scientific), 500 nM of each primer (Lac-F 5’-AGCAGTAGGGAATCTTCCA-3’; Lac-R 5’-CACCGCTACACATGGAG-3’ [30]), and 5 µL of the DNA template. The PCR conditions were: 1 cycle at 95 °C for 5 minutes, followed by 40 cycles at 95 °C for 15 seconds, 58 °C for 20 seconds, 72 °C for 30 seconds and at 80 °C for 30 seconds.

All qPCR were performed using an ABI-PRISM 7500 sequencing detection system (Applied Biosystems, Bridge-Water, NJ, USA). 

For the construction of standard curves, 10-fold dilution series between 10^5^ and 10^1^ copies of genomic DNA from known quantities of genomic DNA extracted from a pure culture of target species were applied for qPCRs. Negative besides “blanks” controls from the DNA extraction kit controls were included in the PCR runs. All amplification reactions were performed in triplicates.

The coefficients for reaction efficiency, calculated as 10^(‒1/slope)^ ‒1, ranged from 98% to 102%, and the correlation coefficients R2 obtained for the standard curve were between 0.98 and 0.99.

The C_t_ (cycle threshold) from each sample was compared with the C_t_ from the standard curve in order to get the number of copies of the 16S rRNA gene in the samples. The minimum limit of detection of the qPCR technique was 1.4 log equivalent cells/mL of human milk. Below that, quantities were considered as not detected.

### 2.9. Statistical Analysis

All statistical analyses were performed using the statistical computing language R. For differences in the human milk microbiota composition, the “adonis” function (PERMANOVA test) was performed using weighted and unweighted UniFrac distances to compare the groups (FOS and placebo) by day (before and after the intervention), using 999 permutations (vegan package). In addition, in order to compare the abundance of individual taxa before and after the intervention trial, ANCOM [31] was applied for repeated measures.Alpha-diversity analyses were performed after applying rarefactions (10,000 sequences/sample) to standardize sequence counts (vegan package). The Mann–Whitney test or Wilcoxon signed ranks test were used to compare the alpha-diversity of independent or dependent samples, respectively.

The Jensen–Shannon distance (JSD) was used to calculate the distribution of the distance between “before” and “after” paired samples by each group (placebo or FOS group). The Mann–Whitney test was used to compare the distributions. 

The Mann–Whitney test or t-test were used to compare *Bifidobacterium* spp. and *Lactobacillus* spp. counts from qPCR between the interventional groups. Wilcoxon signed ranks test for paired samples was used to compare *Bifidobacterium* spp. and *Lactobacillus* spp. counts from qPCR between “before” and “after” supplementation.

Linear regression models were carried out employing the distances between “before” and “after” supplementation as the dependent variable and supplemented group interacting with maternal variables as independent variables. Afterwards, ANOVA was performed to identify statistically significant variables which could explain the variability in these distances. In order to identify the OTUs that could be influencing the distances, linear mixed-effects models were used which employed the relative abundance of OTUs in human milk as the dependent variable and supplemented group and time as factors, adjusted by subjects. ANOVA was performed to compare the models including the interaction effect of the maternal variables or not. For this propose, the “lmer” function (lme4 package) was performed.

A false discovery rate *p* value ≤ 0.01 was considered as significant for analyses with multiple comparisons. A standard *p* value < 0.05 was considered as significant for the analyses.

## 3. Results

**Figure 1:**
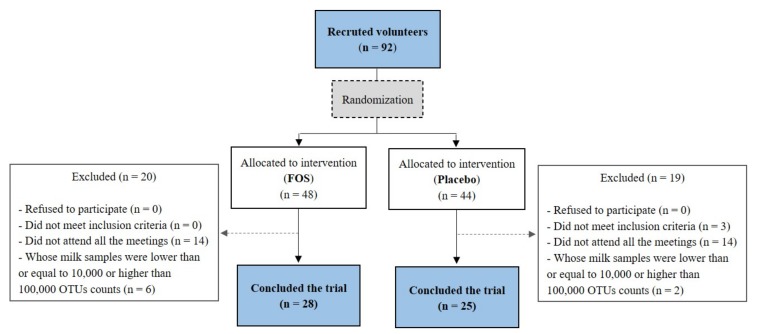
Flow diagram of participant recruitment followed for the study. FOS: Fructooligosaccharide; OTU: Operational Taxonomic Unit. The limits values of 10,000 and 100,000 were chosen arbitrarily based on the graph of OTU counts per sample in order to exclude outliers.

Twenty-eight volunteers from the FOS group and twenty-five volunteers from the placebo group concluded the clinical trial (Figure 1). The clinical characteristics and the estimated nutrient intakes “before” and “after” the supplementation of these volunteers are shown in Table 1 and Table 2, respectively. No differences were found between the placebo and the FOS groups with regard to the clinical characteristics of the volunteers (Table 1). No differences were found for nutrient intakes before the intervention when comparing the groups (*p* > 0.05). After the intervention period, the carbohydrate intake was higher in the FOS group (*p* = 0.006; Table 2). However, the difference was around 22 g, which would not imply in a great variation in terms of nutrients consumed.

Overall tolerance of the FOS and the placebo (maltodextrin) supplements was good. Complaints about bloating were reported by five volunteers in the FOS group. No major side effects were observed.

Regarding the sequences generated by sequencing in the Illumina platform, 3,488,486 *16S rRNA* gene sequences were analyzed after quality filtering, with an average number of high-quality sequences of 32,910 per sample. Reads were classified into 334 OTUs at 97% sequence similarity.

Considering that human milk samples might have a low microbial load, a no-template PCR control and a DNA extraction kit reagent control were sequenced alongside the samples. The genera abundances in the controls were different from the abundances in the milk samples (*p* = 0.001 by the PERMANOVA test comparing controls vs. milk samples; Figure S1). Thus, we concluded that the controls had different profiles to those of the milk samples.

At the beginning of the trial, all the milk samples were clustered together (Figure S2) independently of the group FOS or placebo, indicating no statistical differences between the groups in the human milk microbiota before the intervention (*p* = 0.477 and 0.626 for weighted and for unweighted UniFrac, respectively).

**Figure 2:**
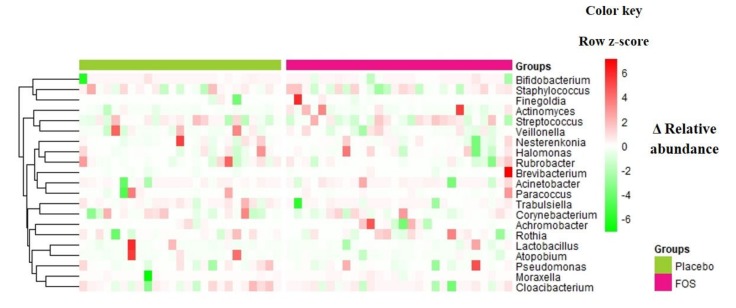
Delta of relative abundances of genera between the day after the intervention period and the day before the intervention, for volunteers from placebo group or FOS group. Columns correspond to differences between relative abundances for each volunteer. Rows correspond to genera with maximum abundance higher than 0.05. Delta was calculated by [(relative abundance of genus at day after supplementation)–(relative abundance of genus at day before supplementation)], therefore, positive values (red) denote an increase of the relative abundance of the taxa along the intervention period, while negative values (green) denote a decrease of the relative abundance of the taxa in the milk samples. The intensity of the colors represents the degree of difference between the means. The values were z-scores transformed by row for ease of visualization of the differences. Rows are clustered by Euclidean distance.

Overall, no statistically significant changes were found in terms of richness and alpha diversity (Figure S3) or relative abundance of genera or OTUs, comparing “before” and “after” supplementation (*p* > 0.05 by ANCOM analysis) in both groups. Figure 2 shows the differences (delta) of the relative abundances between “after” and “before” supplementation of the main genera identified in the human milk samples for each volunteer. 

In contrast with what was expected, no significant differences were found with regard to the effect of the prebiotic supplementation in increasing the *Bifidobacterium* and *Lactobacillus* populations in human milk. These findings inferred from sequencing were later confirmed by qPCR using genera-specific primers, which are more precise for quantifying these genera (Figure S4).

**Figure 3:**
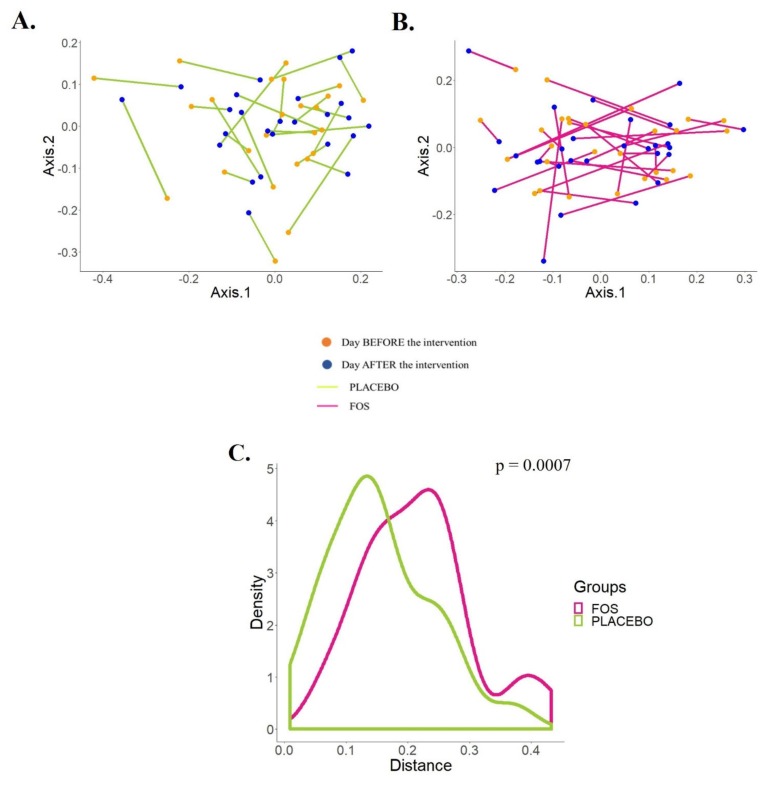
Effects of the supplementation with placebo or fructooligosaccharide (FOS) on the human milk microbiota of each subject. (**A**,**B**) PCoA plots of Jensen–Shannon distance (JSD) shows the effects of the maternal supplementation with placebo (**A**) or FOS (**B**) on the phylogenetic structures of the human milk microbiota. (**C**) Distribution of the distances (JSD) between “before” and “after” supplementation for each subject by group shows statistically significant differences between the placebo and the FOS group (Mann–Whitney test).

At the same time, we explored the trajectory of the human milk microbiota from the starting point (before the supplementation) to the ending point (one day after the end of the supplementation) in the PCoA by the supplemented groups (Figure 3A,B). According to Figure 3C, there is a statistically significant difference between the trajectories of the groups (*p* = 0.0007).

**Figure 4:**
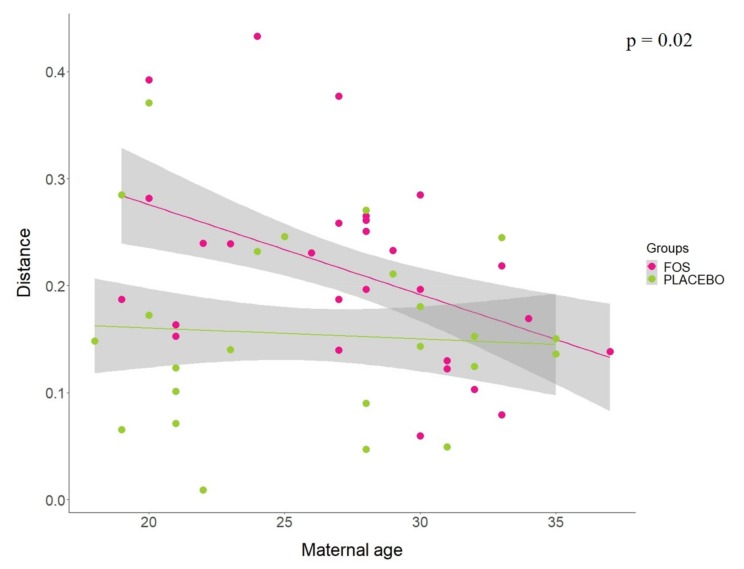
Jensen–Shannon distance between “before” and “after” the supplementation by FOS (pink) or placebo (green) groups, according to the maternal age. Least-squares means was used to compare the slopes of the regression of distance vs. maternal age between FOS and placebo groups.

Additionally, analysis of linear regression models using the Jensen–Shannon distance between “before” and “after” the supplementation by group revealed that the maternal age seems to affect the response for FOS supplementation (*p* = 0.02). Our results indicate that younger mothers were more influenced by the FOS supplementation, whereas older mothers seem to have similar response to the placebo group (Figure 4). No statistical differences were found for other variables tested in the model, including pre-pregnancy BMI (*p* = 0.8528), BMI at 1st month (*p* = 0.4705), antibiotic treatment during pregnancy (*p* = 0.5896), antibiotic treatment during delivery (*p* = 0.7130), ethnicity (*p* = 0.2004), number of children (*p* = 0.3381), baby gender (*p* = 0.1832), and baby feeding at first month (*p* = 0.7154).

Subsequent analyses found statistically significant OTUs belonging to *Bacteroides*, *Shewanella*, *Rheineimera*, *Idiomarina*, *Staphylococcus*, *Streptococcus*, and *Hymenobacter* genera; *Gemellaceae*, *S24*-7 family, and *Bacillales* order, when models with or without the maternal age as an interaction factor were compared. However, the differences between the relative abundances of “after” and “before” the supplementation for these OTUs did not exhibit a pattern related to younger mothers from the FOS group as our previous analysis suggested (Figure S5). Accordingly, the FOS supplementation influenced the human milk microbiota of younger mothers compared to the placebo group. However, the changes found seem to be different for each subject.

## 4. Discussion

In order to investigate the role of the maternal diet supplementation with prebiotics (fructooligosaccharides) during lactation on the human milk microbiota, we performed a randomized, placebo-controlled clinical trial with prebiotic (FOS) or placebo (maltodextrin) supplementation in the maternal diet. 

The beneficial effects of fibers, particularly prebiotics, on the human health are well-established [16,32]. Especially during pregnancy and the lactation period, prebiotics reduce the risk for postpartum weight retention, contributing towards a lower risk for long-term obesity, and consequently, reducing the risk for non-transmissible chronic diseases [16]. Additionally, prebiotics are known for their ability to stimulate beneficial microorganisms in the human gut [32]. In this context, a number of studies suggest that gut bacteria could be transferred to the neonate via breastfeeding since identical strains have been isolated in paired samples of maternal feces and breast milk [33,34].

We expected higher relative abundances of *Bifidobacterium* and *Lactobacillus* in the FOS-supplemented group since these genera are recognized FOS consumers and could be increased in the mother’s gut [35]. However, no differences were observed for these genera between the groups analyzed by either Illumina MiSeq^®^ or qPCR using genera-specific primers. 

Following this line, Shadid et al. [36] investigated the effect of the supplementation with galactooligosaccharide and long chain fructooligosaccharide in women in the last trimester of pregnancy in a randomized, double-blind placebo-controlled study on the maternal and neonate gut microbiota. The authors reported significantly higher *Bifidobacterium* populations in the mother’s feces from the supplemented group than in those from the placebo group. However, no differences were found in the neonate gut microbiota between the placebo or the supplemented group, suggesting that the bifidogenic effect on the maternal gut microbiota was not transferred to the neonates. In our study, the short period of supplementation could be a factor for no statistical differences between the groups. Our recent study identified that long-term maternal dietary habits are more likely to influence the structure of the human milk microbial community, whereas short-term maternal intake induced minor changes in the microbiota composition [37].

We did not find any differences for changes in the relative abundance of the human milk microbiota when comparing the prebiotic-supplemented and the placebo group in our initial analyses. Nevertheless, further analyses suggested that the distances in the PCoA trajectory performed by the FOS group from the initial to the end of the supplementation was longer than those of the placebo group, and maternal age seems to affect this behavior. According to these results, younger mothers seem to be influenced by the FOS supplementation since they have major changes in their microbiota profile after supplementation whereas older mothers have similar distances to the placebo group, indicating that there are no differences between the groups for older mothers. In addition, our results suggest that this influence of the FOS supplementation was different for each subject since no pattern of changes at the end of the intervention were found.

To our knowledge, no previous study has reported the effect of the maternal age on the human milk microbiota; however, the role of age is well-established in terms of modulating the gut microbiota. The metabolic and physiologic patterns of each stage of life are described as the main reasons for changes in the gut microbiota [38]. Nonetheless, the age range of the volunteers in this study was from 18 to 37 years old, which would not imply substantial physiologic changes. It is possible that differences in dietary or lifestyle patterns could be related to the differential response to FOS supplementation, since the dietary intake may vary substantially during the life-course [39,40].

In this context, Parsons et al. [39] identified marked diet changes in the majority of subjects evaluated over eight years (from age 33 to 42). A greater proportion of cohort members reduced their potato chip consumption and increased their fruit and salad consumption. Similarly, Mishra et al. [40] investigated longitudinal changes in dietary patterns during adult life (at age 36 to 53). In their study, the consumption of meat, potatoes and sweet foods in women was reduced.

Consequences of different dietary patterns throughout life may be related to influences on the gut microbiota [41], which have been extensively related to inter-individual variations in response to dietary interventions [42,43].

Marked inter-individual variation in the gut microbiota of individuals who received non-digestible carbohydrates was reported by Walker et al. [42]. The authors’ findings suggested that differential responses were dependent on the initial composition of an individual’s gut microbiota. In addition, Griffin et al. [42] suggested that prior dietary practices, based on chronic calorie restriction with adequate nutrition or without dietary restrictions, might impair responses to dietary interventions, which would require the introduction of diet-responsive bacterial lineages present in other individuals. Similarly, an in vitro study evaluated the effect of a prebiotic mixture on two different fermentation systems, varying in their nutritional availability. The authors reported differential responses of the gut microbiota to the same prebiotic formula, including some OTUs within the same genus, which responded to the prebiotic in opposite ways [44].

Apart from differences in the composition of the gut microbiota, immunological effects of diet interventions could also be associated with the presence of individual taxa. According to Martínez et al. [45], a short-term intake of whole grains induced compositional alterations of the gut microbiota. However, subjects with greater improvements in plasma Interleukin-6 levels harbored significantly higher proportions of *Dialister* and a lower abundance of *Coriobacteriaceae*. In this context, immunological changes resulting from prebiotic supplementation seem to be transferred to the human milk [46,47], which could influence the local microbiota [48].

In our study, we describe for the first time the influence of the prebiotic supplementation on the human milk microbiota. We could not obtain samples of maternal feces to investigate whether the maternal gut microbiota would be related to the differential microbial changes observed in milk for the FOS supplementation. Nevertheless, although we cannot rule out confounding factors, the change in microbial directories by prebiotic supplementation warrants further investigation. Neither did we evaluate the immunological compounds or oligosaccharides present in the human milk, which would be interesting to explore if changes in these factors could be also associated with changes in the milk microbiota composition. A longer intervention period would be desirable, and this would possibly lead to more perceptible changes in the human milk microbiota.

The factors that influence the human milk microbiota, particularly concerning the maternal diet, are still largely unknown, and further investigations are required to better understand the possible benefits of maternal diet intervention on maternal and infant health to be validated in the clinical practice afterwards.

## 5. Conclusions

We demonstrated that manipulating the human milk microbiota through prebiotics (FOS) is possible, and that maternal age can affect this response. However, the pattern of changes seems to be individual-dependent. Further studies are required for a deeper understanding of whether other factors, especially the individual gut microbiota, immunological compounds or oligosaccharides present in the human milk could be related to the differentiated responses towards FOS supplementation.

## Figures and Tables

**Table 1:** Clinical and demographic characteristics of the volunteers who concluded the clinical trial, according to the groups (*n* = 53).

Variable	Groups		
	FOS	Placebo	*p*-value **
	*n* = 28	*n* = 25	
**Maternal Age (Years)**	28 (23–31)	28 (21–31)	0.469
Race			0.597
Black/Brown	12 (42.9)	12 (48)	
White	16 (57.1)	13 (52)	
Family income estimated (USD/month) *	770.4 (462.5–770.4)	462.5 (462.5–770.39)	0.458
Duration of pregnancy (weeks)	39 (38–40)	39 (38–40)	0.611
Maternal antibiotic treatment			
during pregnancy	6 (21.4)	7 (28)	0.814
during delivery	11 (39.3)	10 (40)	1.000
Alcohol drinking during pregnancy	3 (10.7)	1 (4)	0.687
Smoking during pregnancy	2 (7.1)	1 (4)	1.000
BMI before pregnancy (kg/m^2^)	23.0 (20.7–25.2)	22.6 (20.9–24.5)	0.929
Maternal weight gain over pregnancy (kg)	11.2 (8–14.1)	11.9 (8.3–16.6)	0.497
Anesthesia			1.000
No anesthesia	7 (25)	3 (12)	
Local	13 (46.4)	14 (56)	
Pudendal block	0	0	
Epidural	0	4 (16)	
Spinal	8 (28.6)	3 (12)	
BMI at day 30 after delivery (before supplementation)	23.6 (21.7–27.4)	24.5 (22.3–26.9)	0.879
BMI at day 50 after delivery (after supplementation)	24.1 (21.8–27.7)	24.6 (22.1–26.5)	0.973
Baby gender			0.940
Male	17 (60.7)	14 (56)	
Female	11 (39.3)	11 (44)	
Birth weight (g)	3205 (2933.7–3563.7)	3210 (2977.5–3425)	0.979
Birth length (cm)	48.5 (47.1–50)	49 (47–50)	1.000
Infant diet before the supplementation			0.145
Exclusively breast milk	23 (82.1)	24 (96)	
Breast milk + formula	5 (17.8)	1 (4)	
Infant diet at day 50 after delivery after the supplementation			0.145
Exclusively breast milk	21 (75)	21 (84)	
Breast milk + formula	7 (25)	4 (16)	
Infant Δ weight over the intervention period (g)	655.0 (458.7–832.5)	792.5 (578.7–900.0)	0.212

Data presented as median (interquartile range) or n (%). * Family income estimated by Brazilian Economic Classification Criteria (Brazilian Criteria) 2016. ** *p*-values were calculated to compare FOS and placebo groups using the Mann–Whitney test for continuous data and the chi-square test for categorical data. BMI: Body Mass Index (kg/m^2^). NA: not applicable.

**Table 2:** Estimated nutrients intake “before” and “after” the supplement intervention, by groups.

	Placebo			FOS		
	Before	After	p-values *	Before	After	p-values *
Energy (kcal)	2091 (1950–2328)	2295 (1956–2608)	0.691	2266 (2105–2503)	2161 (1960–2338)	0.418
Carbohydrates (g)	299 (256–341)	272 (257–319)	0.731	355 (296–398)	250 (227–338)	0.006
Proteins (g)	76 (67–97)	91 (68–110)	0.560	80 (67–91)	84 (74–98)	0.866
Fat (g)	71 (55–83)	72 (62–82)	0.874	72 (63–81)	76 (70–84)	0.797
Saturated fatty acids (g)	23 (20–30)	16 (11–18)	0.691	26 (21–29)	29 (21–31)	0.797
Monounsaturated fatty acids (g)	20 (16–26)	19 (17–26)	0.560	22 (17–25)	25 (21–29)	0.884
Polyunsaturated fatty acids (g)	11 (9–13)	28 (22–33)	0.300	15 (13–18)	16 (13–18)	0.537
Total dietary fibers (g)	22 (19–23)	22 (18–25)	0.596	26 (21–35)	22 (17–26)	0.099

* *p*-values were calculated by Wilcoxon signed ranks test for paired samples to compare the nutrients intake between the days for each group.

## Supplementary Materials

The following are available online at https://www.mdpi.com/2072-6643/12/4/1081/s1, Figure S1: Comparison of bacterial profiles between controls and all human milk samples collected before and after the clinical intervention. Figure S2: Principal Coordinate Analysis (PCoA) of the human milk microbiota of samples at the day before the beginning of the clinical trial for each group (FOS or placebo). Distances measured by unweighted (A) or weighted (B) UniFrac. No statistical differences were found between the groups by the PERMANOVA test. Figure S3: Alpha diversity and richness values of milk samples, by supplemented groups, for each group (FOS and placebo), before and after the supplementation. The Alpha diversity is measured by Shannon (A) and Observed (B) indexes. The richness is measured by Chao1 index (C). Figure S4: Box plot of the distribution of data obtained by quantitative PCR (qPCR) before and after 20 days of supplementation with fructooligosaccharide (FOS group) or maltodextrin (placebo group). (A) *Bifidobacterium* spp. levels in human milk samples collected before and after supplementation for FOS group (pink) or placebo group (green). (B) *Lactobacillus* spp. levels in human milk samples collected before and after supplementation for FOS group (pink) or placebo group (green). Figure S5: Heatmap for differences between the relative abundances of “after” and “before” the intervention with placebo or FOS. Only statistically significant OTUs are displayed, according to ANOVA test for two linear mixed-effects models; one considering the relative abundance of OTUs in human milk as the dependent variable and supplement group and time as the factors and other including the interaction effect of the maternal age.
