# Supplementary figures and images for: Response of the Human Milk Microbiota to a Maternal Prebiotic Intervention Is Individual and Influenced by Maternal Age
Source: Nutrients. 2020 Apr 13;12(4):1081. doi: 10.3390/nu12041081 (PMC7230887; doi:10.3390/nu12041081)

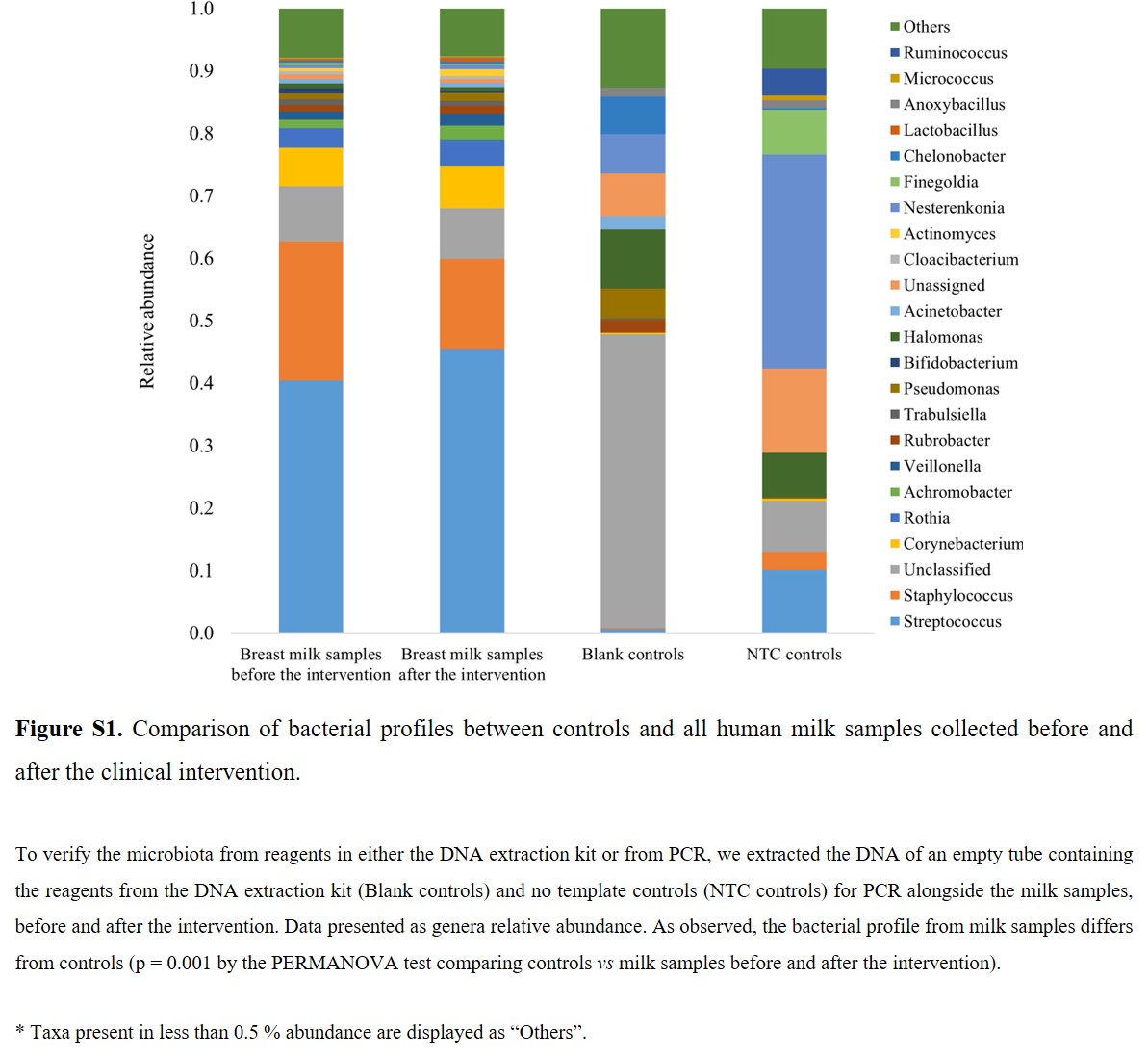

Supplement: Supplementary file 1 [file nutrients-12-01081-s001.zip › Figure_S1.jpg]

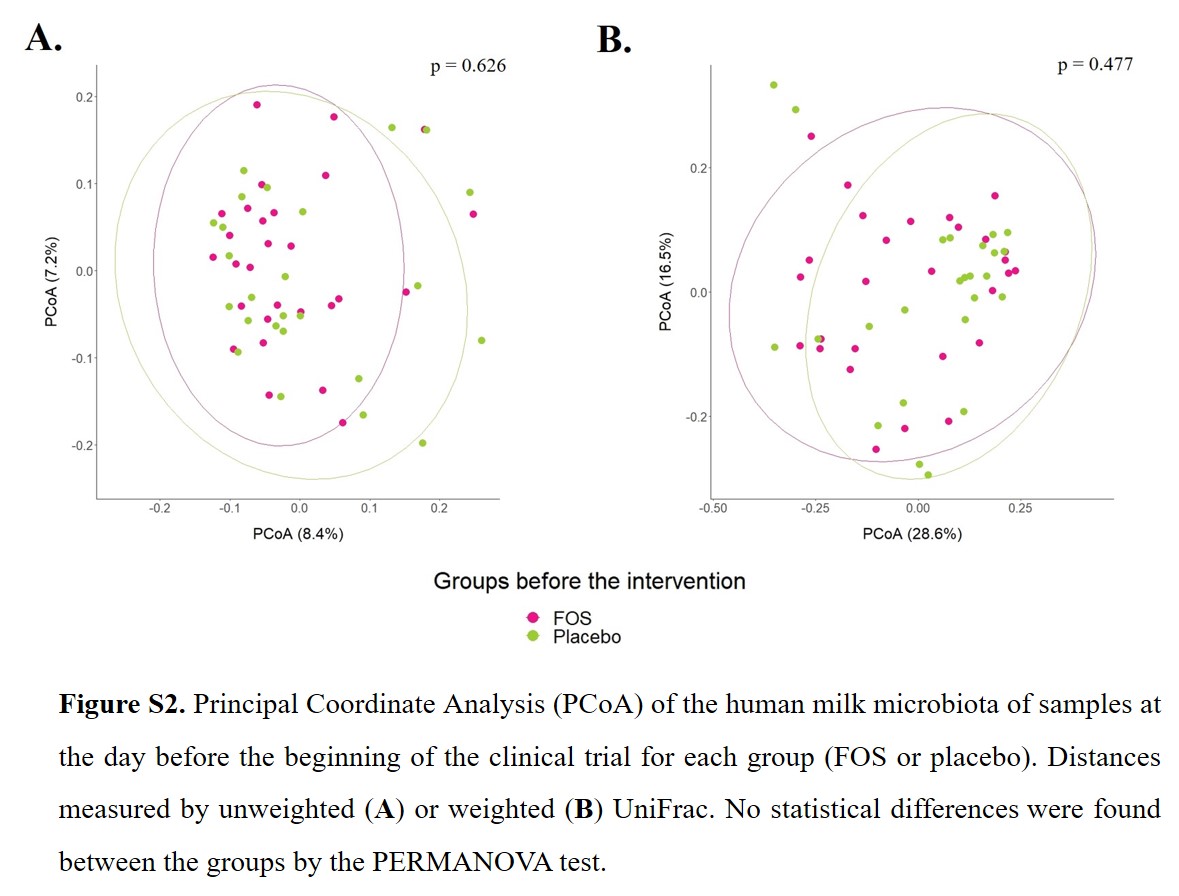

Supplement: Supplementary file 1 [file nutrients-12-01081-s001.zip › Figure_S2.jpg]

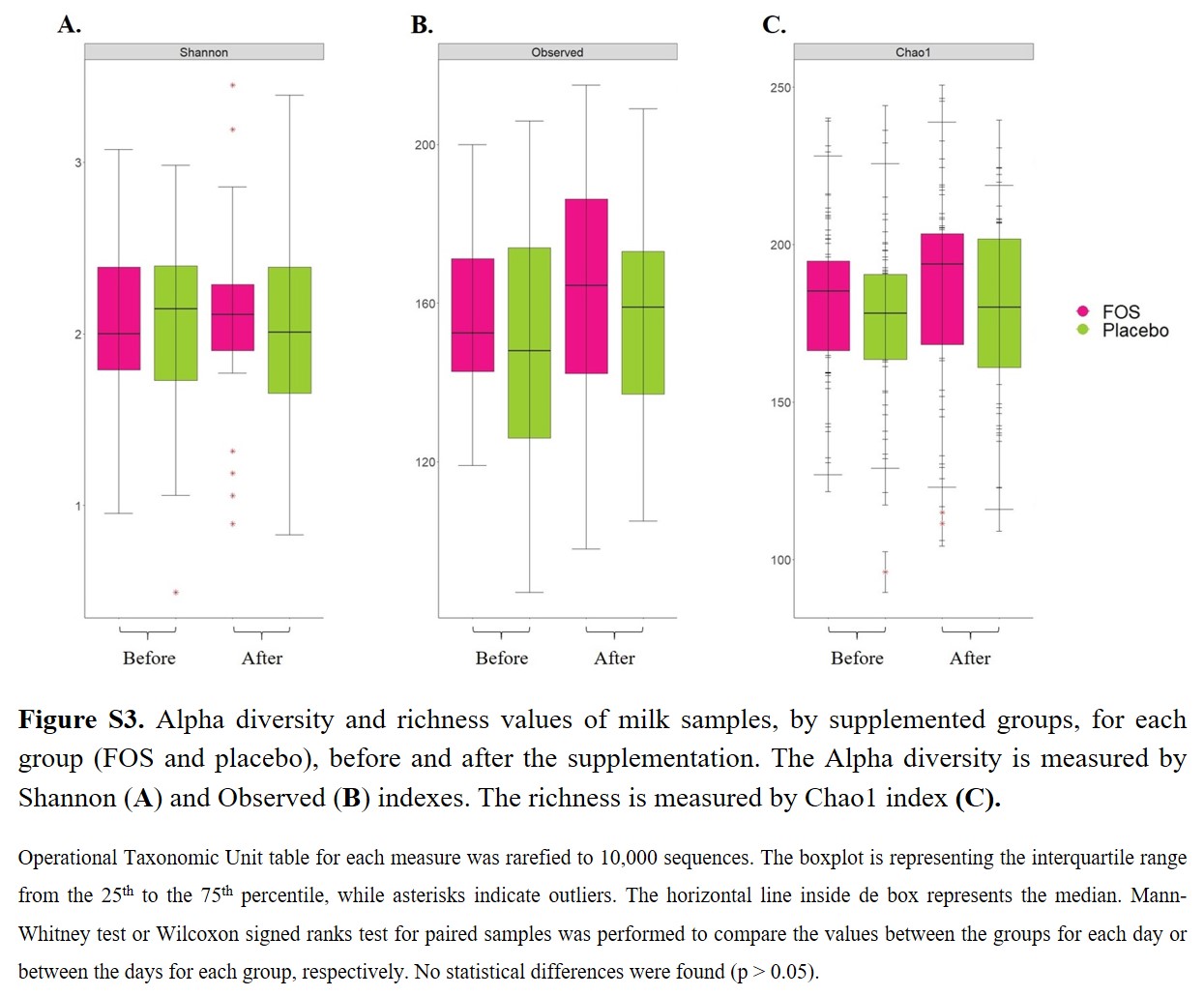

Supplement: Supplementary file 1 [file nutrients-12-01081-s001.zip › Figure_S3.jpg]

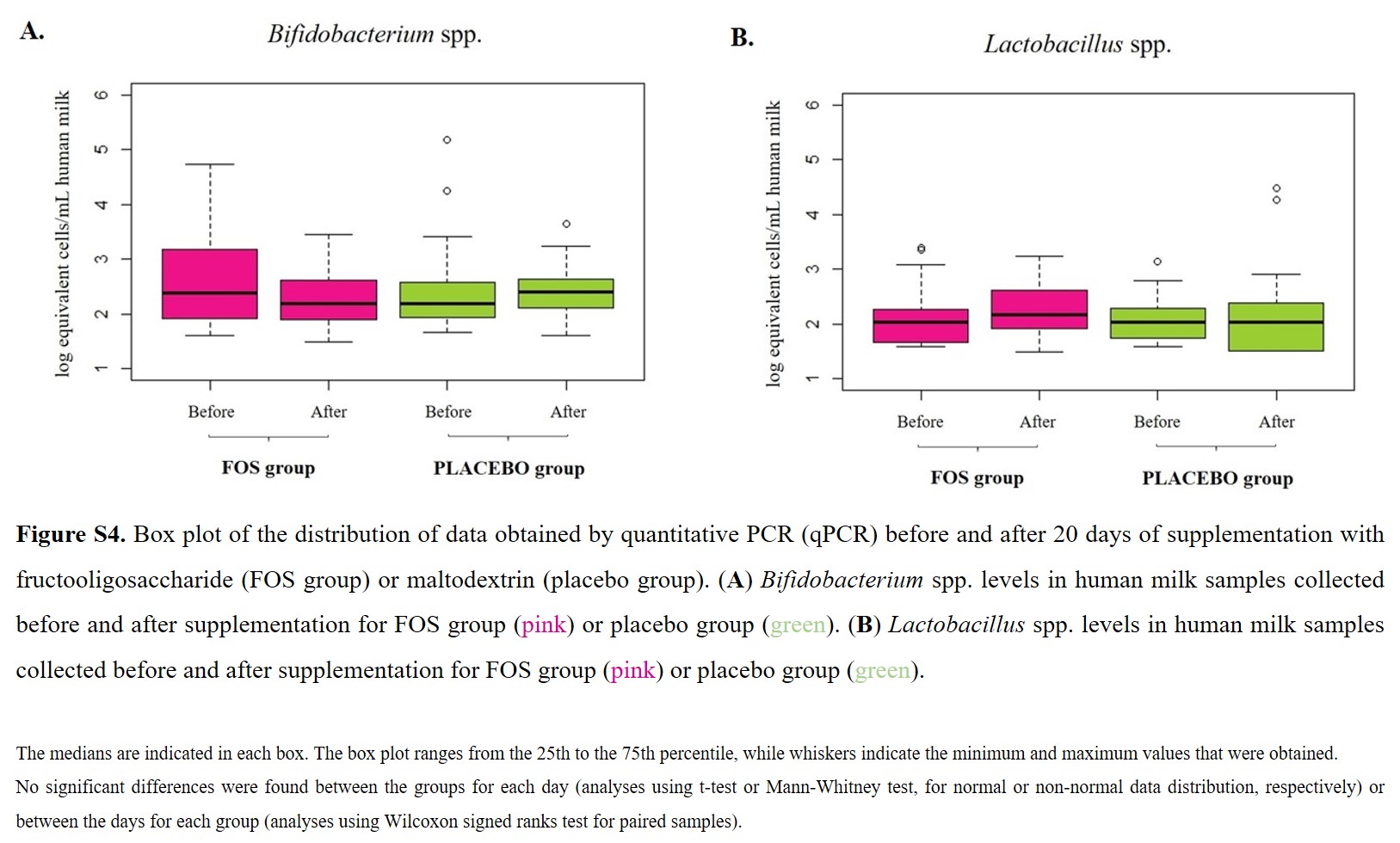

Supplement: Supplementary file 1 [file nutrients-12-01081-s001.zip › Figure_S4.jpg]

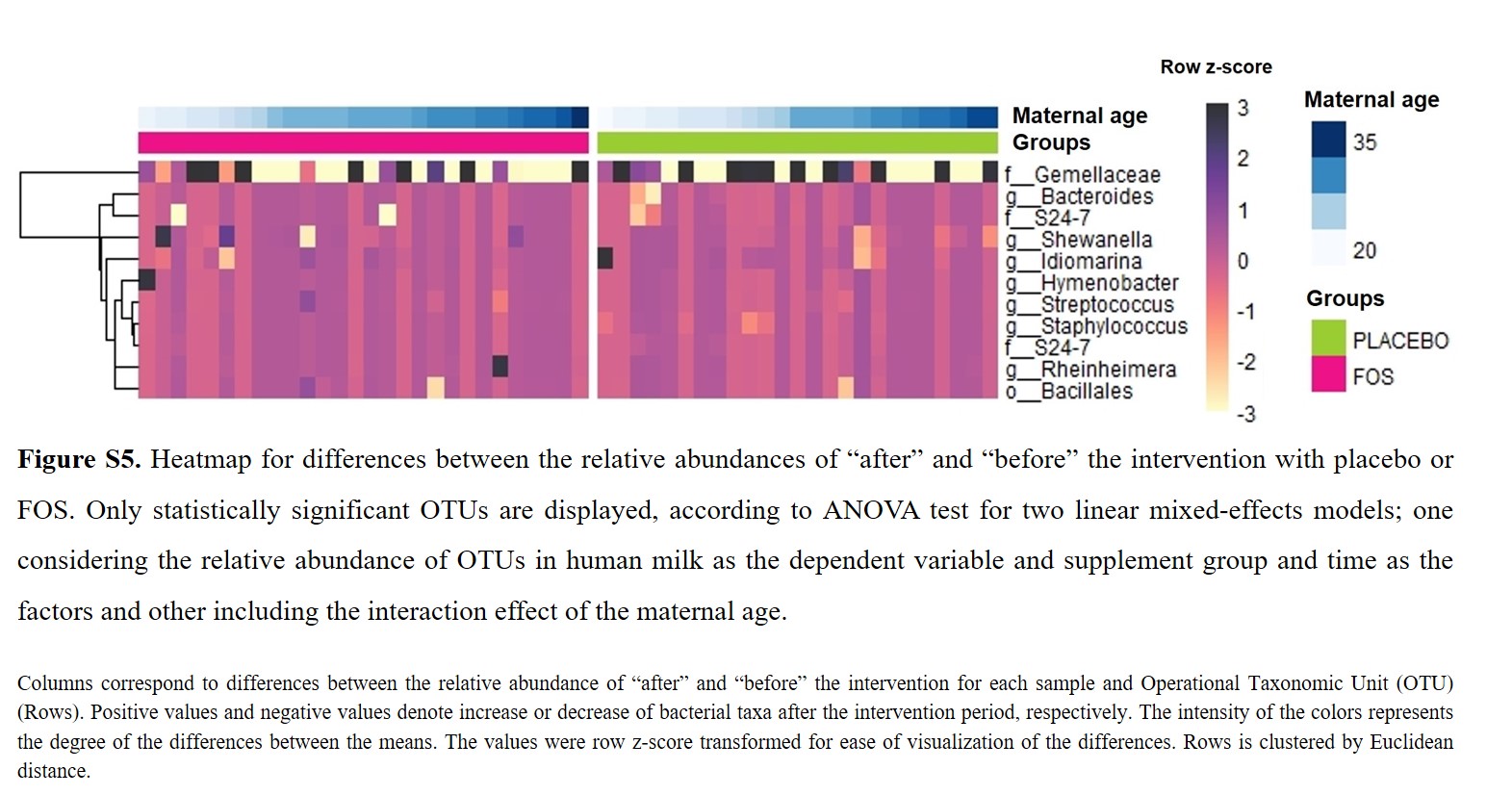

Supplement: Supplementary file 1 [file nutrients-12-01081-s001.zip › Figure_S5.jpg]
